# Graphene-based dental adhesive with anti-biofilm activity

**DOI:** 10.1186/s12951-017-0322-1

**Published:** 2017-12-12

**Authors:** Agnese Bregnocchi, Elena Zanni, Daniela Uccelletti, Fabrizio Marra, Domenico Cavallini, Francesca De Angelis, Giovanni De Bellis, Maurizio Bossù, Gaetano Ierardo, Antonella Polimeni, Maria Sabrina Sarto

**Affiliations:** 1 Research Center for Nanotechnology Applied to Engineering of Sapienza University (CNIS), Sapienza University of Rome, Piazzale Aldo Moro 5, 00185 Rome, Italy; 2 Sapienza Nanotechnology & Nano-science Laboratory (SNN Lab), Sapienza University of Rome, Piazzale Aldo Moro 5, Rome, 00185 Italy; 3 Department of Aerospace, Electrical and Energy Engineering, Sapienza University of Rome, Via Eudossiana 18, Rome, 00184 Italy; 4 Department of Biology and Biotechnology “Charles Darwin”, Sapienza University of Rome, Piazzale Aldo Moro 5, Rome, 00185 Italy; 5 Department of Dentistry and Maxillo-Facial Sciences, Unit of Pediatric Dentistry Sapienza University of Rome, Viale regina Elena 287a, Rome, 00161 Italy

## Abstract

**Background:**

Secondary caries are considered the main cause of dental restoration failure. In this context, anti-biofilm and bactericidal properties are desired in dental materials against pathogens such as *Streptococcus mutans*. To this purpose, graphene based materials can be used as fillers of polymer dental adhesives. In this work, we investigated the possibility to use as filler of dental adhesives, graphene nanoplatelets (GNP), a non toxic hydrophobic nanomaterial with antimicrobial and anti-biofilm properties.

**Results:**

Graphene nanoplatelets have been produced starting from graphite intercalated compounds through a process consisting of thermal expansion and liquid exfoliation. Then, a dental adhesive filled with GNPs at different volume fractions has been produced through a solvent evaporation method. The rheological properties of the new experimental adhesives have been assessed experimentally. The adhesive properties have been tested using microtensile bond strength measurements (µ-TBS). Biocidal activity has been studied using the colony forming units count (CFU) method. The anti-biofilm properties have been demonstrated through FE-SEM imaging of the biofilm development after 3 and 24 h of growth.

**Conclusions:**

A significantly lower vitality of *S. mutans* cells has been demonstrated when in contact with the GNP filled dental adhesives. Biofilm growth on adhesive-covered dentine tissues demonstrated anti-adhesion properties of the produced materials. µ-TBS results demonstrated no significant difference in µ-TBS between the experimental and the control adhesive. The rheology tests highlighted the necessity to avoid low shear rate regimes during adhesive processing and application in clinical protocol, and confirmed that the adhesive containing the 0.2%wt of GNPs possess mechanical properties comparable with the ones of the control adhesive.

## Background

Resin composites can be considered the most used class of materials in dental restorations due to their improved aesthetic quality. Furthermore, materials used in this type of restoration are bonded to the teeth hard tissues via adhesives, enabling a non-invasive caries removal approach [1]. Despite these positive aspects, the use of polymeric composites maintain some main drawbacks such as polymerization shrinkage and high bacteria adhesion affinity [2]. In presence of micro-cavities between the healthy tissues and the dental restoration, bacteria can easily access to the cured dental tissues through cavities at the tooth-restoration interface [3]. Caries at the margin of a restoration are still considered the main cause of composite dental restorations failure [4]. Thus, in recent years, there has been an increasing clinical and academic interest in the development of anti-biofilm adhesives [5].

Biofilms are the first responsible of the dental caries etiopathogenesis and in general, of periodontal diseases. The conventional anti-biofilm strategies are generally focused on developing substrates able to inhibit the bacteria attachment and colonization [6].

Hence, once the formation of the biofilm is complete, its external viscoelastic matrix become the main defence mechanism against the conventional antibiotic drugs. Its disruption can occur only by macro-mechanical removal (i.e. tooth brushing) or by using drugs able to digest the biofilm matrix [7].

In order to develop anti-biofilm dental adhesives, the basic approach is to embed antibacterial agents in restorative materials [8]. Nanomaterials [9–13], chlorhexidine [14], quaternary ammonium compounds [15–19] or methacrylate-based monomers [20, 21] have been extensively investigated as antimicrobial components of dental resins. The antimicrobial mechanism mainly lies on killing pathogens through cations release [22, 23], cytoplasmic membrane mechanical disruption [24, 25] and reactive oxygen species (ROS) production [26, 27].

Recently, graphene based materials (GM) such as graphene oxide (GO) [28] or graphene nanoplatelets [29] (GNP) have been tested in solution against dental pathogens demonstrating the possibility to use them in dental materials. The antimicrobial and anti-biofilm effect of these materials can be ascribed to three main different mechanisms originated by the direct interaction between GM and bacteria cells [30]. The first mechanism is related to the 2D nanostructures ability to wrap the cells. Thus, materials such as GO, reduced-GO (rGO) or GNP are able to induce mechanical stress and preventing nutrient uptake [31]. The second interaction mechanism involve the nanostructure sharp edges. Due to the high aspect ratio and low dimensions, nanostructures behave as nano-knives, penetrating and disrupting the cell membrane [32]. Finally, the third and the most widely acceptable mechanism is based on the oxidative stress production [33]. However, it is of particular relevance with respect to biofilm growth inhibition, the possibility to reduce the bacteria adhesion on the substrate. In general biofilm adhesion over a substrate is enhanced by the presence of reactive structural defects over its surface [34]. Therefore, from this point of view, it is important to highlight that there is a crucial difference between GO and GNPs since the presence of oxygen-containing functional groups on the basal plane enhances bacterial adhesion over GO-substrate much more than over GNP-substrate. Moreover, the presence of such functional groups in GO led to its ability to produce a surplus of reactive oxygen species (ROS) [35, 36], which can contributes to the antimicrobial effect of GO with respect to GNPs, but it can be also correlated to a higher cytotoxicity [37]. Liao et al. [38] demonstrated that the blood compatibility and cytotoxicity of graphene-based materials is proportional to the oxygen content. Other studies confirm that GO and rGO main drawback is the presence of these reactive oxygen functional groups, considered the main cause of toxicity, as demonstrated by Olteanu et al. [39].

In particular, Lee et al. [40] demonstrated that different dental adhesive monomers are responsible of oxidative stress associated toxicity in fibroblast and pulp cells. Under the hypothesis that biologically active additives can be incidentally released from dental materials, Demirci et al. [41] demonstrated the possibility to have excess of ROS production, with consequent induction of oxidative stress and genotoxicity by dental adhesives. Moreover, it is worth to consider that the majority of ROS-based antimicrobial mechanisms proposed nowadays can affect the saliva redox equilibrium, increasing the risk of a decrease in the natural oral immune system defences [42].

Finally, GNP are multilayer graphene nanoplatelets possessing biocidal properties based on their ability to act as nano-knives; moreover, their basal planes are similar to the ones of the graphene sheet, which is free of functional groups able to promote unwanted bacterial adhesion like in GO or rGO. Indeed, it is demonstrated that 2D single layer graphene possesses anti-adhesion properties against biofilm [43] and does not show any biocidal activity [44]. However, despite its large-scale use in academic and scientific research, 2D graphene is nowadays not suitable for large-scale production due to the high costs and technological limits. Thanks to their low cost, highly scalable, non-toxic and non-oxidizing production process, GNP can represent a viable solution for large-scale development of anti-biofilm devices. Since they possess the basal plane properties typical of the 2D graphene, at a lower cost and without its technological problems [45], GNP can be a valid alternative to graphene, being, at the same time, characterized by sharp edges and large surface-to-volume ratio. This allows the mechanical interaction of GNP with the bacterial cell walls resulting in strong antimicrobial properties. Moreover, GNP does not induce ROS, due to the presence of carbon as the only elementary constituent [46].

In this work, we propose the use of GNP as filler of an experimental dental adhesive, in order to obtain an adhesive with antimicrobial and antibiofilm properties. The main innovation of our work consists in the particular production process of the new adhesive. This result in GNPs dispersed uniformly, without agglomerations inside the polymer and partially exposed over the adhesive surface. Actually, GNPs emerging on the surface allow the adhesive to exert an antimicrobial and antibiofilm activity against *S. mutans.* We prove that the porosity of the dental substrate, combined with the proper rheological properties of the new material, plays a crucial role in the mechanical and antimicrobial properties of the experimental adhesive. Here we demonstrate that the proposed adhesive has a biocidal local mechanism, thus preserving the oral cavity microenvironment without producing any additional oxidative stress.

## Methods

### Materials

Ethanol, for HPLC, gradient grade (≥ 99.8%) was purchased from Sigma Aldrich, Milan, Italy. The commercial dental adhesive composed by BisGMA, HEMA, dimethacrylates, ethanol, water, photo-initiator system and containing silica nanoparticles at the 10%wt was purchased from 3 M, Italy (in the following referred to as “control adhesive”). Graphite Intercalated Compound (GIC) purchased from Sigma Aldrich, Milan, Italy, were used as precursor material for graphene nanoplatelets (GNP) production.

#### GNPs production

GNPs are produced by using GIC as precursor as described in [29]. Briefly, the GIC undergoes a thermal driven expansion in a muffle furnace, leading to the formation of wormlike expanded graphite (WEG). The temperature and duration of the thermal shock was set at 1050 °C for 30 s.

WEG were then dispersed in ethanol and exfoliated through tip sonication, using a sonotrode (Sonics & Materials Vibracell VCX750) working at 20 kHz for 20 min, with ultrasound amplitude set at 70%, and operating at 15 °C in pulsed mode (1 s off, 1 s on). A colloidal suspension of GNP in ethanol is obtained. As reported by Rinaldi and co-workers [47], the produced GNPs are composed by a number of staked graphene planes comprised between a few and several tens, with a resulting thickness comprised between ~ 1 and ~ 25 nm and typical lateral dimensions comprised between a few hundreds of nanometers and a few microns.

#### Experimental dental adhesive production

The experimental adhesive consists of a dental commercial adhesive (named sample A in the following) filled with GNP (in the following referred to as “experimental adhesive”). In order to obtain a uniform dispersion of GNP without formation of agglomerations, the GNPs suspension produced as described above was added to the commercial adhesive. Three different samples of experimental adhesive were produced, with different weight concentrations of GNPs, i.e. 0.1%wt (sample A01), 0.2%wt (sample A02) and 0.5%wt (sample A05).

**Fig. 1:**
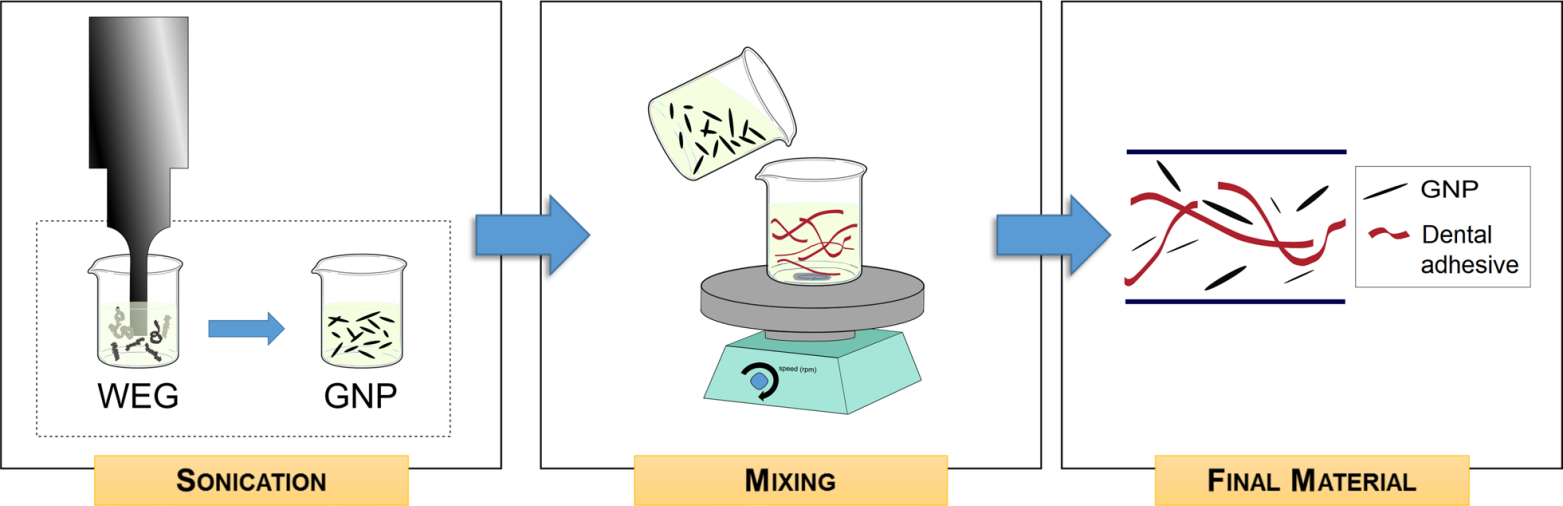
Schematics of the experimental dental adhesive production process. The wormlike expanded graphite (WEG) undergoes tip sonication to obtain GNP. Then, the obtained suspension is mixed with the standard dental adhesive. Finally, the obtained material consists in a good dispersion of the filler nanoparticles among the polymer chains

Controlled slow-rate evaporation of the solvent in excess at room temperature and at room pressure is performed through mechanical stirring, for a time comprised between 6 and 12 h, depending on the nanofiller concentration. The evaporation process is stopped when the mixture has a total over weight with respect to the reference adhesive of the 120%, so that the resulting mixture is characterized by a Newtonian rheological behaviour, with measured viscosities comprised between 0.01 and 0.1 Pas, enabling a uniform application of the antimicrobial dental adhesive in dental cavity. The so obtained mixture is then applied over the substrate and subjected to air flushing of the free-surface with a controlled air-flux pressure of 0.2 bar (according to the reference commercial adhesive data-sheet). Finally, UV/vis polymerization follows (Fig. 1).

#### Field emission-scanning electron microscope analysis

All the composites produced were characterized through field emission-scanning electron microscope (FE-SEM). The obtained experimental adhesives were spotted onto three classes of substrates with different porosities in order to investigate how the substrate porosity affects the nanofiller distribution over the adhesive surface. Glass coverslips (p_0_) were used as non-porous reference. Anodic aluminium oxide membrane disks with uniform pore size of 20 nm (p_20_) and of 150 nm (p_150_) were used as porous substrates.

Constant volumes of the liquid adhesives were spotted onto the substrates using a pipette. Successively, air blowing on the free-surface of the dental adhesive with a controlled air-flux pressure of 0.2 bar was performed. Finally, the samples underwent UV/vis polymerization for 20 s at intensity greater than 800 mW/cm^2^.

Before imaging, to avoid charging of the non-conductive surface under investigation, the samples underwent metallization: a 20-nm Cr film is sputtered on the fracture surfaces using a sputter coater (Q150T, Quorum Technologies Ltd., Laughton, UK). FE-SEM investigation was carried out using a Zeiss Auriga available at Sapienza Nanotechnology and Nanoscience Laboratory, operated at voltages varying between 2 and 5 keV, depending on the sample type.

#### Rheology measurements

Rheological characterizations of both the commercial and experimental adhesives were carried out using a rotational rheometer (Anton Paar, MCR302) operated in steady shear state mode. The measurements were performed at 23 °C using a Peltier controlled temperature hood to avoid material evaporation during the test. The entire set of tests was performed by using a 50 mm plate–plate geometry. Apparent viscosity was measured in the range of shear rates from 1 1 to 100 1/s, using a 0.4–0.6 mm gap between the plates. The sample set A was tested as purchased, while the rheological properties of the experimental adhesives were measured after production.

#### Microtensile bond strength test

Extracted adult permanent molars were used after obtaining informed consent from the donors. All teeth were stored in distilled water at 4 °C for a maximum of 3 months, and the storage medium was replaced every 3 days to minimize deterioration. Twenty-four molars were randomly divided into 4 groups, corresponding to the A, A01, A02 and A05 sample sets. While fully hydrated, each molar was cut immediately below the occlusal pit and fissure by using a diamond blade (Secotron 200, Remet, Italy) under water cooling. The dentin surfaces were subsequently wet-polished using 600–1200–4000 grit silica paper to create a uniform and flat surface. All the groups were treated with 3 M Scotchbond Universal Etchant (30–40% phosphoric acid) for 15 s. Then the surfaces were rinsed and air-dried. Subsequently, adhesive systems were applied for 15 s and dental cotton rolls were used to remove the excess adhesive. A light-curing machine was used to light cure the surfaces for 20 s at an intensity of ≥ 800 mW/cm^2^.

Four 2-mm thick layers of the 3 M ESPE Z100 MP restorative paste were applied onto the previously polymerized adhesive layer and light cured for 20 s.

After treatment, all the teeth were further sectioned following the ISO/TS 11405 standard into resin-dentin sticks with an average cross-sectional area of 1 mm × 1 mm by using a diamond blade (Secotron 200, Remet, Italy) under water cooling. The specimens were then stored in deionized (DI) water at 37 °C for 24 h. Finally, the produced samples underwent micro-tensile bond strength test (µ-TBS), performed by using the universal testing machine Instron 3366 (Instron, USA). The beams were fixed on a jig using a cyanoacrylate adhesive and then subjected to tensile forces at a crosshead speed of 1 mm/min.

#### Strain and growth conditions

*Streptococcus mutans* ATCC 25175 was grown in Brain Heart Infusion broth (BHI, DIFCO) at 37 °C.

#### Antimicrobial tests

The analysis was performed in 96-well microtiter plates. A controlled volume (200 μl) of each produced dental adhesive was spotted in each well. Afterward, 5 min evaporation was performed in static conditions in order to eliminate residuals of the ethanol. Finally, the samples were polymerized using a curing lamp for 20 s from the top and 20 s from the bottom. Moreover, samples were washed after polymerization with DI water and underwent sterilization under UV for 3 h. A suspension of an overnight growth culture of *S. mutans* was diluted to 1 × 10^8^ cells/ml into fresh BHI. Next, 20 μl of that suspension was used to inoculate the 96-well microtiter plates. Following 1 h incubation at room temperature under sterile conditions, 180 μl of BHI broth were added to each well containing the adhesives. Microtiter plates were then incubated for 24 h at 37 °C under static conditions. Aliquots of samples were withdrawn, diluted and then spread onto BHI agar plates. After incubation at 37 °C the capacity of the bacteria to form colonies was manually measured by counting the number of Colony Forming Units (CFU).

#### Biofilm growth on teeth

Extracted adult permanent molars were cut at two different heights in order to obtain 5 mm discs and wet-polished using 600–1200–4000 grit silica paper to create two uniform and flat surfaces.

Subsequently, the lateral enamel surfaces were wet-polished with 600 grit silica paper to create cubic teeth samples with exposed dentin on the six surfaces and finally wet-polished using 1200–4000 grit silica paper. Then, one surface was used to hold the sample while five of the six surfaces were treated with 3 M Scotchbond Universal Etchant (30–40% phosphoric acid) for 15 s. Then rinsed and air-dried. Subsequently, A and A02 adhesive systems were applied for 15 s, dental cotton rolls were used to remove the excess adhesive. A light-curing machine was used to light cure the surfaces for 20 s at an intensity of ≥ 800 mW/cm^2^. After UV-sterilization, samples were placed in a 12-well microtiter plate and an overnight culture of *S. mutans* was diluted to 5 × 10^6^ cells/ml into BHI with 5% sucrose. Next, 3 ml of such bacterial suspension was added to each well and biofilm was grown on teeth covered by adhesive containing or not GNPs for 3 and 24 h at 37 °C under static conditions. For FE-SEM analysis, samples were washed with distilled water, fixed with 2% glutaraldehyde for 1 h and then dehydrated through serial incubations with 30, 50, 70, 90 and 96% ethanol. Afterwards, teeth were attached on Si wafers and sputtered with gold. A Crystal Violet (CV, Sigma) assay was performed to quantify biofilm formation on teeth samples after 24 h of growth. Briefly, teeth samples were washed twice with sterile water, fixed for 15 min at 65 °C and then stained with 0.3% CV for 15 min. After several washings with sterile water, teeth were air-dried and then photographed. Finally, 96% ethanol was used to dissolve CV bound to teeth biofilm and absorbance at 600 nm was then read for CV quantification.

#### Evaluation of oxidative stress

*Streptococcus mutans* 24 h old-biofilms were grown directly on glass coverslip coated by control or A02 adhesives by placing them in 3 ml of BHI supplemented with 5% sucrose. Briefly, coverslips were prepared by spotting the dental materials onto glass surfaces. Afterward, an air flow was applied in order to let the adhesive volatile phases evaporate and samples were polymerized as described above. Biofilm was scraped from glass coverslips into a 1.5 ml microtube containing 0.3 ml of 1× phosphate-buffered saline pH 7.4 (PBS) and then washed. After that, cells were loaded with 10 µM 2′,7′-dichlorodihydrofluorescein diacetate (H2DCFDA, Thermoscientific) for 45 min at 37 °C and then washed twice with PBS. Flow cytometric analysis was used to assess the production of free intracellular radicals by using a FACS Calibur system (BD Biosciences, San Jose, CA). Biofilm treated with 5% H_2_O_2_ for 30 min was used as positive control for ROS production.

#### Statistical analysis

Data are presented as mean ± SD, and Student’s *t* test or one-way ANOVA analysis (GraphPad Prism 5.0 software) were used to determine the statistical significance between experimental groups. Post-hoc Dunnett’s procedure for comparing treatments with the A control group was performed. Statistical significance was defined as *p < 0.05, **p < 0.01, and ***p < 0.001.

## Results

### FE-SEM and rheology measurements analysis

**Fig. 2:**
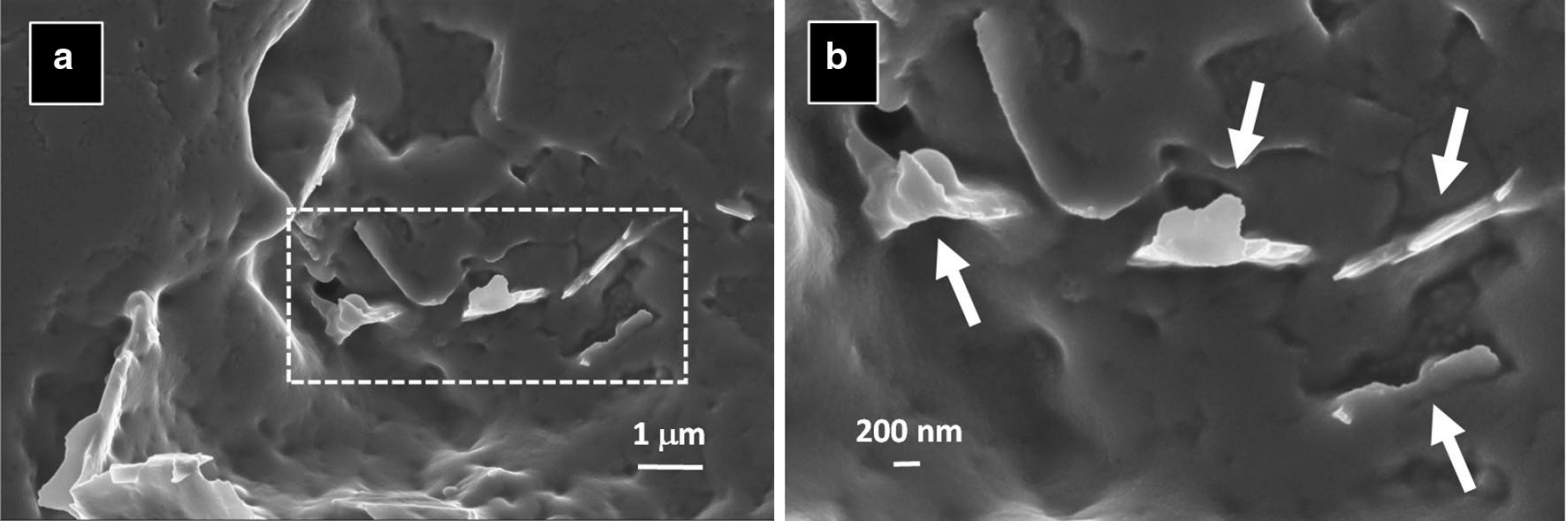
FE-SEM top-view micrographs showing the detail of GNP interaction with the polymer matrix: **a** low magnification and **b** higher magnification of the polymer-nanostructures interface. GNPs (pointed out by white arrows) are well integrated into the adhesive, thus demonstrating the uniform dispersion achieved

FE-SEM analysis demonstrated a good integration between GNPs and polymer in the produced experimental adhesive. Figure 2a, b show that GNPs are well dispersed and fully embedded in the matrix, with sharp edges emerging from the polymer free surface.

In order to analyse the effects of substrate porosity on the GNPs distribution over the adhesive surface, a FE-SEM analysis has been performed on the produced adhesives spotted, spread and polymerized on the three substrates having different porosities, as described above.

**Fig. 3:**
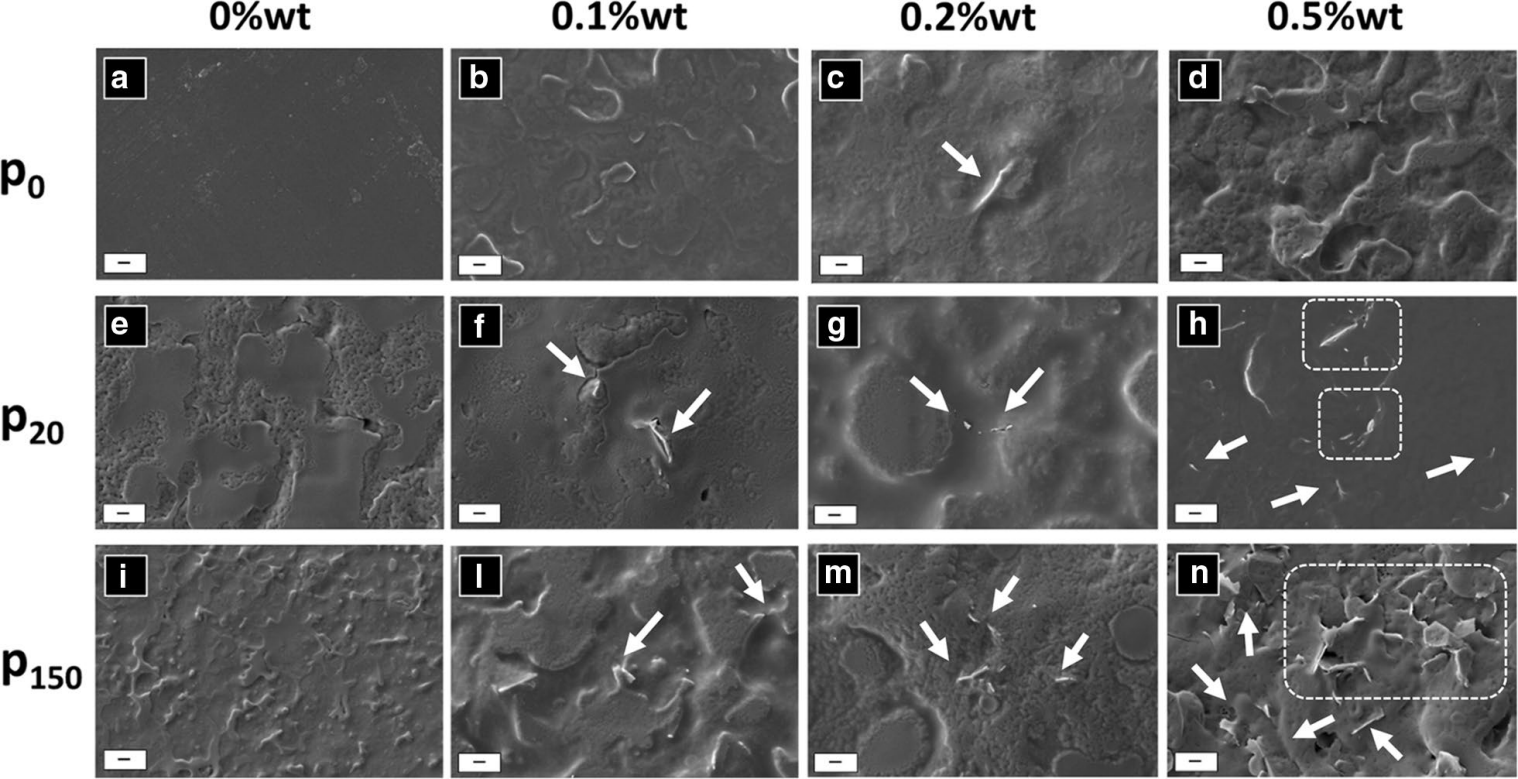
FE-SEM top-view micrographs showing different magnifications of the tested sample sets on different substrates. The micrographs illustrate the differences in nanofiller distribution and exposition over the adhesive surface (GNPs are pointed out by the white arrows and dotted frames): **a**, **e**, **i** represent the control adhesive on different substrates ( p_0_, p_20_ and p_150_). GNPs are embedded in correspondence of the p_0_ substrate (**b**–**d**); the nanofillers are well dispersed and exposed over the surface of adhesives filled with at 0.1 and 0.2%wt, applied on the substrates p_20_ and p_150_ (**f**, **g**, **l**, **m**); GNPs are exposed over the surface of the adhesive filled with at 0.5%wt, more densely when applied over the substrate with larger porosity p_150_ (**n**) than over the substrated with smaller porosity p_20_ (**h**). Scale bar 1 µm

The FE-SEM micrographs of the tested materials show that GNPs are well dispersed in the matrix of the specimens A01 and A02 on all the substrates, as reported in Fig. 3b–c, f–g, l–m. By contrast, in sample A05 we notice an evident agglomeration effect of the nanofiller (Fig. 2d, h, n. Moreover, FE-SEM analysis reveals a strong difference in terms of GNPs exposure over the adhesive surface, depending on the substrate porosity and filler concentration. In particular, in correspondence of a flat substrate, GNPs result barely exposed for all concentrations. Nevertheless, increasing the pore dimensions (i.e. 20 and 150 nm), the amount of GNPs edges exposed over the adhesive surface depends on the GNPs concentration. Specifically, in samples filled at 0.1%wt and at 0.2%wt, we observe a uniform exposition of the GNPs over the adhesive surface applied on both substrates with smaller and larger porosity (Fig. 3f–l, g–m, respectively). On the contrary, in the sample filled at 0.5%wt, GNPs are very well exposed over the adhesive surface, only in the specimen produced using the substrate with larger porosities (Fig. 3h–n).

In parallel, in order to correlate the differences in concentration with the differences in mechanical properties of the materials, a rheological analysis has been performed.

**Fig. 4:**
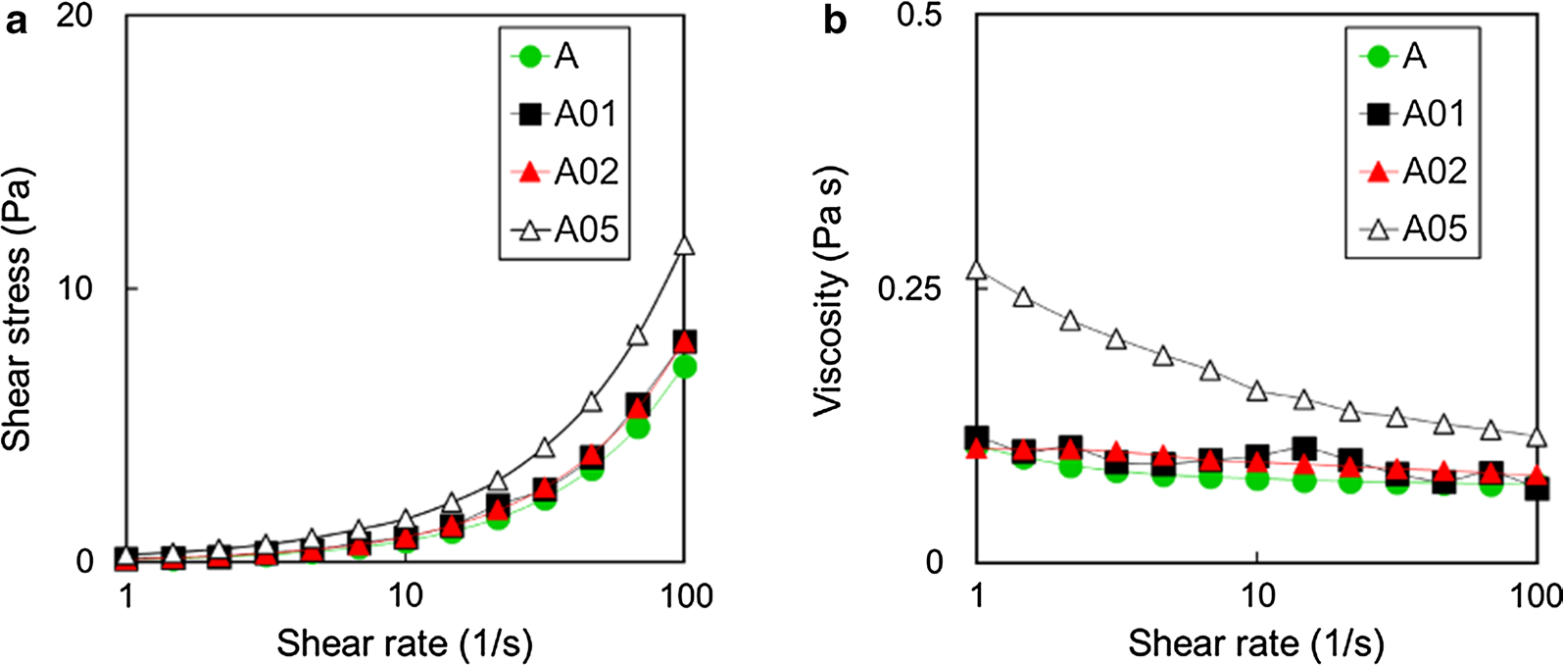
Shear stress (**a**) and viscosity (**b**) of the produced samples measured at room temperature (23 °C), as function of the shear rate

Figure 4a, b show the measurements of shear stress and viscosity for the samples at 23 °C after 60 min of solvent evaporation. The results highlight the differences between the samples A01, A02 and the sample having the highest concentration of GNPs (i.e. A05). Both A01 and A02 show a rheological behaviour comparable with the one of the reference adhesive A (green circles in Fig. 3), corresponding to a Newtonian behaviour within the shear rate range investigated. Although similar values of shear stress are recorded for all analysed samples (Fig. 4a), the experimental adhesive A05 displays a higher viscosity with respect to the other specimens (Fig. 3b).

#### Microtensile bond strength tests

The results of the microtensile bond strength (μTSB) are reported in Table 1. Different weight concentration of GNPs were tested; the control group has μ-TBS corresponding to 31.44 ± 3.40 MPa.

**Table 1:** List of the produced samples adhesion properties and Dunnett’s adjusted p value

Sample set	GNP concentration (%wt)	Microtensile bond strength (MPa)	p value	Premature failure (%)	Number of tested specimens
A	–	31.44 ± 3.40	–	0	18
A01	0.1	31.17 ± 2.54	0.9871	0	18
A02	0.2	31.55 ± 3.89	0.9988	0	18
A05	0.5	29.07 ± 2.05	0.0003	77.8	4

The mean μ-TBS for the A01 and A02 samples corresponds to values almost similar to the control and no composite detachment prior to testing was observed. By contrast, at the higher tested filler loadings (i.e. A05), the 77.8% of specimens resulted in failure prior to testing; these samples were included in statistical calculations following the procedure described by Roulet et al. [48]. Briefly, the lower bond strength of the respective tested set was attributed to each failed specimen. In this way, it has been possible to statistically quantify the effect of increasing filler concentrations on the material adhesive properties. Moreover, the Dunnett’s adjusted p value was calculated using the tooth as the statistical unit as in Beloica et al. [49]. Three samples for each tooth were tested and the average values used for statistical analysis. Our results demonstrate no statistical difference between the experimental dental adhesives A01 and A02 when compared with the control group (Table 1).

#### Anti-biofilm and cells vitality tests

**Fig. 5:**
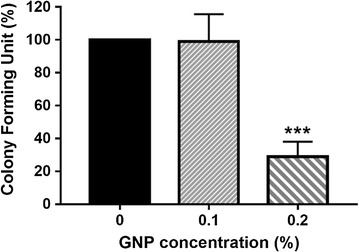
CFU results of *S. mutans* in contact with the produced sample sets. Error bar indicates standard deviation

Based on the previous results, the antibacterial properties of experimental adhesives were evaluated in the A01 and A02 samples, thus excluding the material showing failure prior to adhesion testing (Fig. 5). After 24 h of incubation with dental adhesives, A02 sample demonstrates high antibacterial activity; only 28% of *S. mutans* are able to survive to the treatment with A02 adhesive. By contrast, A01 sample induces no effect on *S. mutans* survival (Fig. 5).

**Fig. 6:**
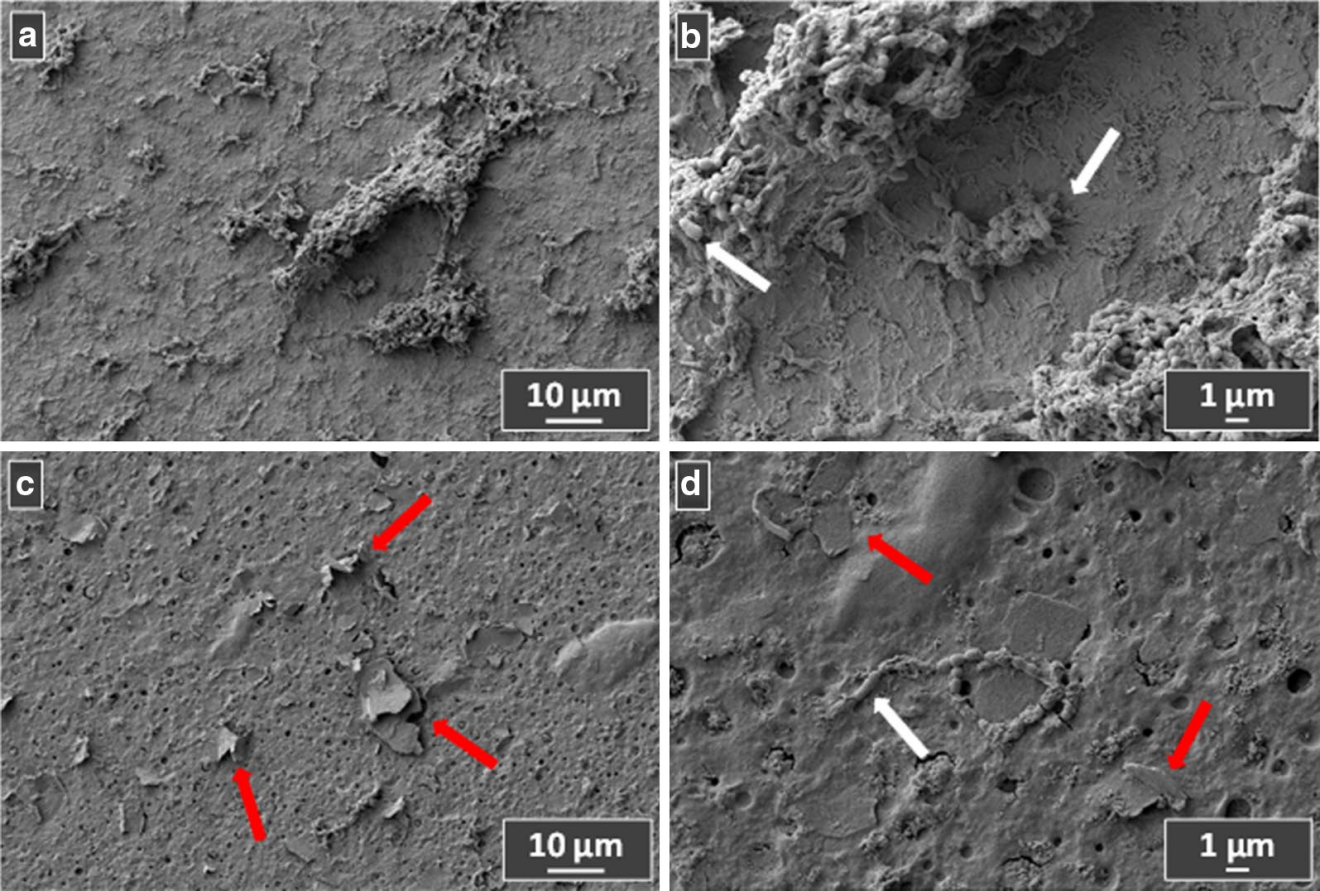
**a**, **b** Two different magnitudes of the 3 h-growth of *S. mutans* biofilm on the teeth coated with the control adhesive A. **c**, **d** Represent two different magnitudes of the 3 h-growth of *S. mutans* biofilm on teeth-coated by experimental adhesive A02

**Fig. 7:**
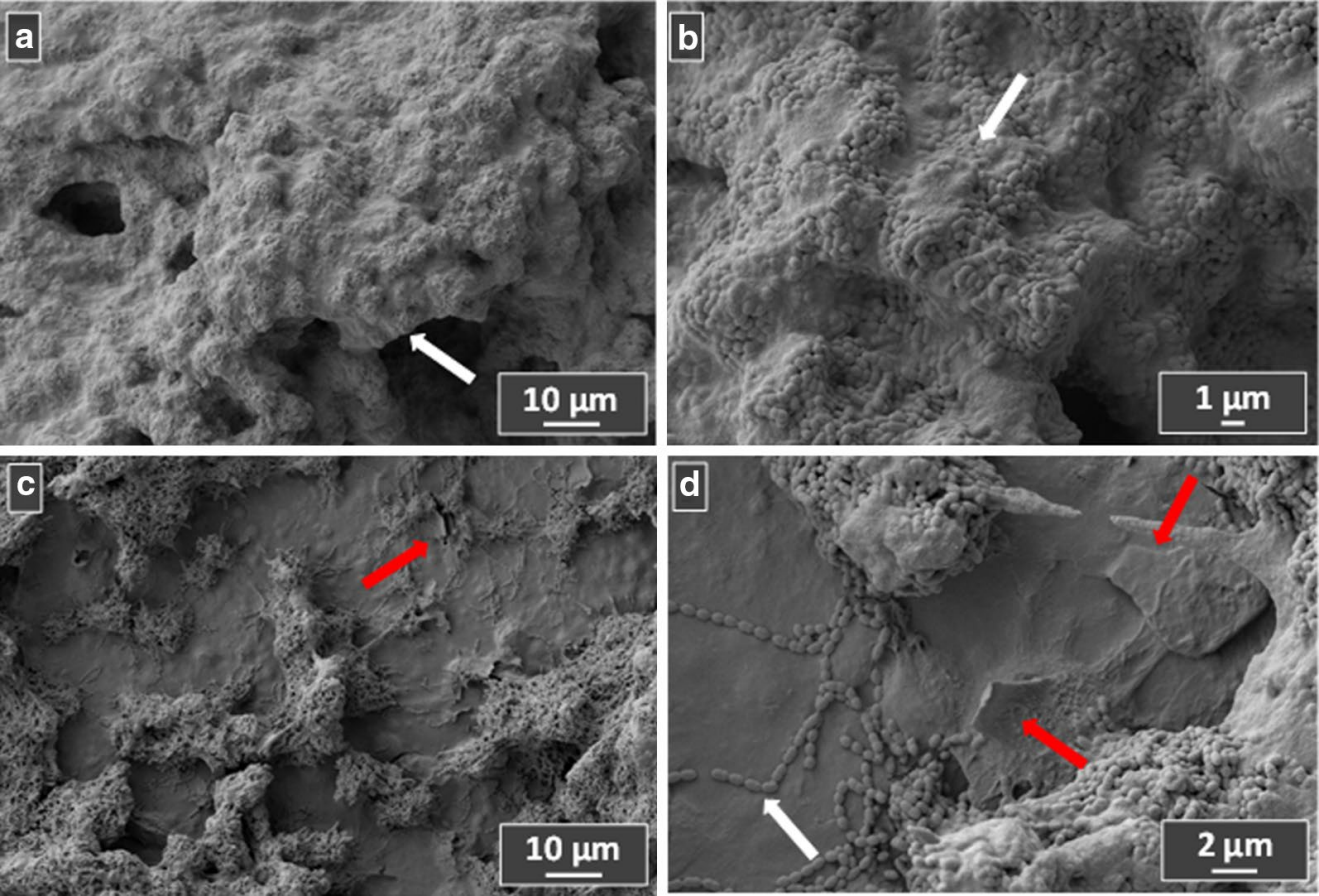
**a**, **b** Two different magnitudes of the 3 h-growth of *S. mutans* biofilm on the teeth coated with the control adhesive A. **c**, **d** Represent two different magnitudes of the 3 h-growth of *S. mutans* biofilm on teeth-coated by experimental adhesive A02

Due to its adhesion properties together with the anti-*Streptococcus* action, the ability to inhibit biofilm adhesion and growth is evaluated in the case of the A02 sample. For this purpose, the A02 experimental adhesive and the control adhesive A are applied on teeth samples, and used as substrates for biofilm growth. Figures 5 and 6 show the FE-SEM images of *S. mutans* biofilm after 3 h and 24 h growth, respectively. White arrows indicate the bacteria cells, while the red arrows represent the GNPs appearing on the top of the material. In Fig. 5a, b the 3 h growth on the control adhesive is shown: it is clearly visible the initial biofilm formation after bacterial adhesion. By contrast, in the experimental adhesive (Fig. 5c, d) cell adhesion is limited, as confirmed by the reduced amount of bacteria. Figure 7 illustrates the biofilm growth after 24 h: a massive biofilm formation/maturation of *S. mutans* cells is clearly observed on the control adhesive (Fig. 7a, b). Noteworthy, from the comparison of Figs. 6a, c, it is possible to observe a lower extent of biofilm growth over the A02 sample. Moreover, bacterial cells are not able to colonize those areas where they are in direct contact with GNPs emerging from the adhesive surface (Fig. 7c, d, red arrows).

**Fig. 8:**
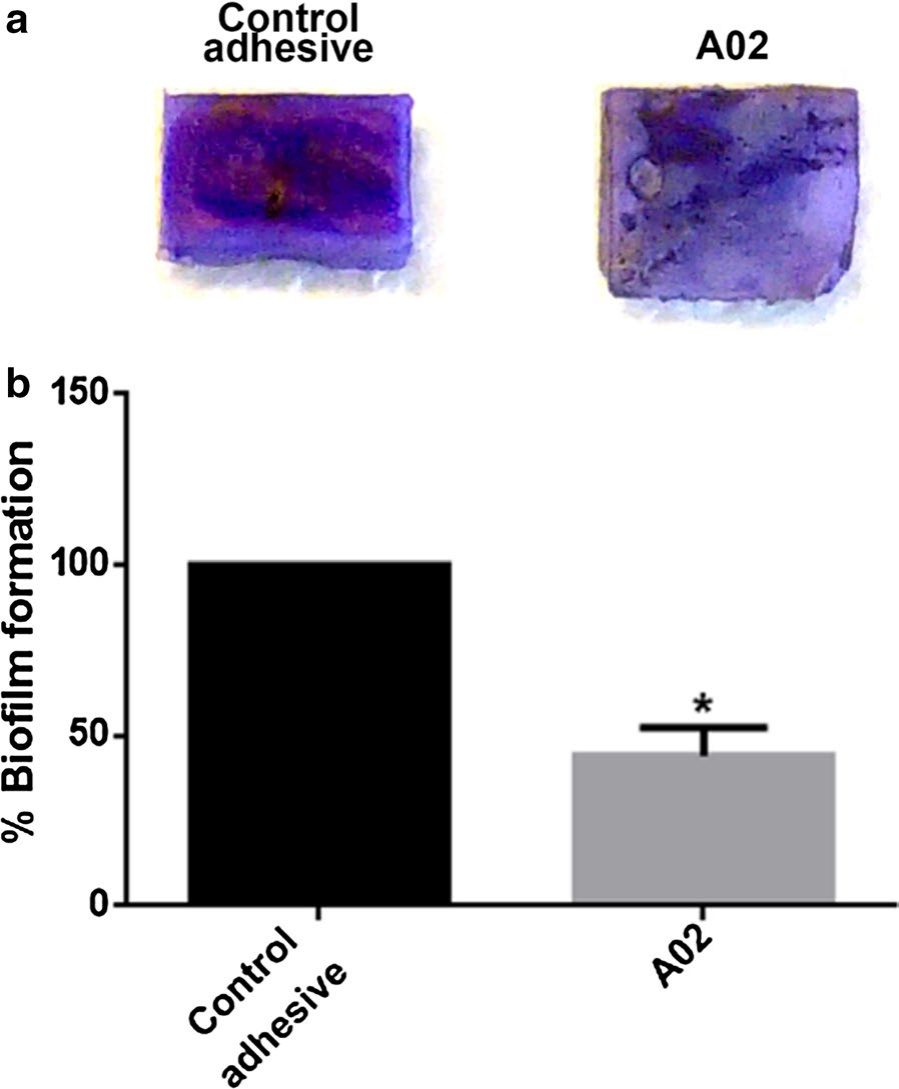
**a** Photographs of control adhesive- and A02-covered teeth stained with CV. **b** Biofilm biomass analysis on CV stained teeth. Histograms are the mean of three independent experiments. Error bars indicate SD and Student’s t test was used to assess statistical significance (*p < 0.05 with respect to control adhesive)

This inhibitory action of GNP-containing adhesive is confirmed by the CV assay, a dye specific for biofilm biomass. Figure 8a shows the pictures of CV stained tooth surfaces covered by dental adhesives containing or not GNP, and demonstrates clearly the inhibition of biofilm formation exerted by the experimental sample; A02-covered teeth result less stained in comparison with control adhesive A. Indeed, the A02 adhesive is able to reduce biofilm formation on teeth by 56%, with respect to the control adhesive (Fig. 8b).

**Fig. 9:**
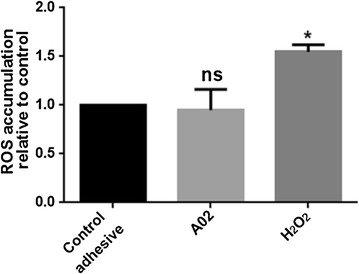
Cytosolic ROS quantification by measuring the dichlorofuorescein diacetate (H_2_DCFDA) probe activation through ROS generation in *S. mutans* biofilm grown on adhesives containing or not GNPs. Data are expressed as ROS accumulation relative to commercial adhesive sample. As a positive control is shown ROS amount of hydrogen peroxide-treated biofilm. Statistical analysis was performed by one-way ANOVA method coupled with the Bonferroni post-test (ns not significant; *p < 0.05 with respect to control adhesive)

To investigate a possible mechanism of action of A02 adhesive against the biofilm mass, reactive oxygen species (ROS) are evaluated by utilizing the fluorescent probe H_2_DCFDA. The accumulation of ROS in the *S. mutans* biofilm was analyzed by incubating the cells with the fluorescent dye dihydrorhodamine 123. This compound accumulates inside the cells and is oxidized by ROS to the corresponding fluorescent cromophore. FACS analysis revealed no differences in ROS amount in cells grown on both types of adhesives (Fig. 9). Biofilm mass challenged with hydrogen peroxide was used as a positive control.

## Discussion

GNPs as fillers of polymer adhesive represent a promising innovative solution to prevent and inhibit *S. mutans* proliferation in oral cavity due to the mechanical interaction between the nanostructured material emergent from the polymer surface and the cell wall. The results highlight a difference in the way the nanostructures protrude from the adhesive surface, depending both on the pores dimensions and on the adhesive viscosity (and filler concentration). In the case of non-porous substrates, FE-SEM images show that GNPs are completely embedded inside the polymer matrix at all the concentrations. Increasing the pore size at a fixed concentration and viscosity, the polymeric phase of the adhesive tends to penetrate inside the substrate pores due to capillarity, whereas the 2D-nanostructures (having lateral dimensions much larger than the substrate porosity) protrude from the adhesive surface. This process is helped by the air flow which is applied during the adhesive application and curing phases.

Niem et al. [50] demonstrated that unfilled polymer systems with shear thinning behaviour could possess high adhesive performances using higher air-blowing pressures and forcing adequate penetration into dentine tubules. In case of filled adhesives, the air blowing phase plays a fundamental role in order to maintain a uniform dispersion of the nanofiller in the polymeric adhesive and to obtain a rough surface of the adhesive. A maximum pressure of 1 bar has been used in order to maintain a uniform dispersion of the nanofiller and avoid losses of material.

FE-SEM images demonstrate that in the sample filled at the 0.5%wt, GNPs agglomerations occur without relevant differences between the three substrates with different porosity (i.e. no porosity, 20 nm porosity and 150 nm porosity). This effect is confirmed by the rheological behaviour at high shear stresses, where the applied stress is able to increase the probability of particle to particle interactions and, more in general, of agglomeration occurrence, leading to a higher viscosity.

Low viscosity of the polymer is a desirable property of dental adhesives as it facilitates the penetration into the dentine tubules and, consequently, it increases the interlocking mechanism efficiency.

Rheology measurements demonstrated that at low concentrations (i.e. A01 and A02) the material possesses good GNPs dispersion and it shows a rheological behaviour very similar to the one of the reference sample set A. This was also confirmed by the FE-SEM micrographs, in which we did not observed the formation of agglomerates for both the 0.1 and 0.2%wt samples.

When all the produced materials underwent μ-TBS, samples containing GNP concentration of 0.5%wt demonstrated not acceptable failure rates prior to testing. In fact, the GNPs, as revealed by FE-SEM analysis, agglomerate at this concentration suggesting that the resultant composite is of lower mechanical strength. Indeed, the A05 set showed 77.8% failure prior to testing. On the contrary, in the A01 and A02 sample sets no failure prior to testing occurred and the μ-TBS results were strongly comparable with the ones of the control group.

**Fig. 10:**
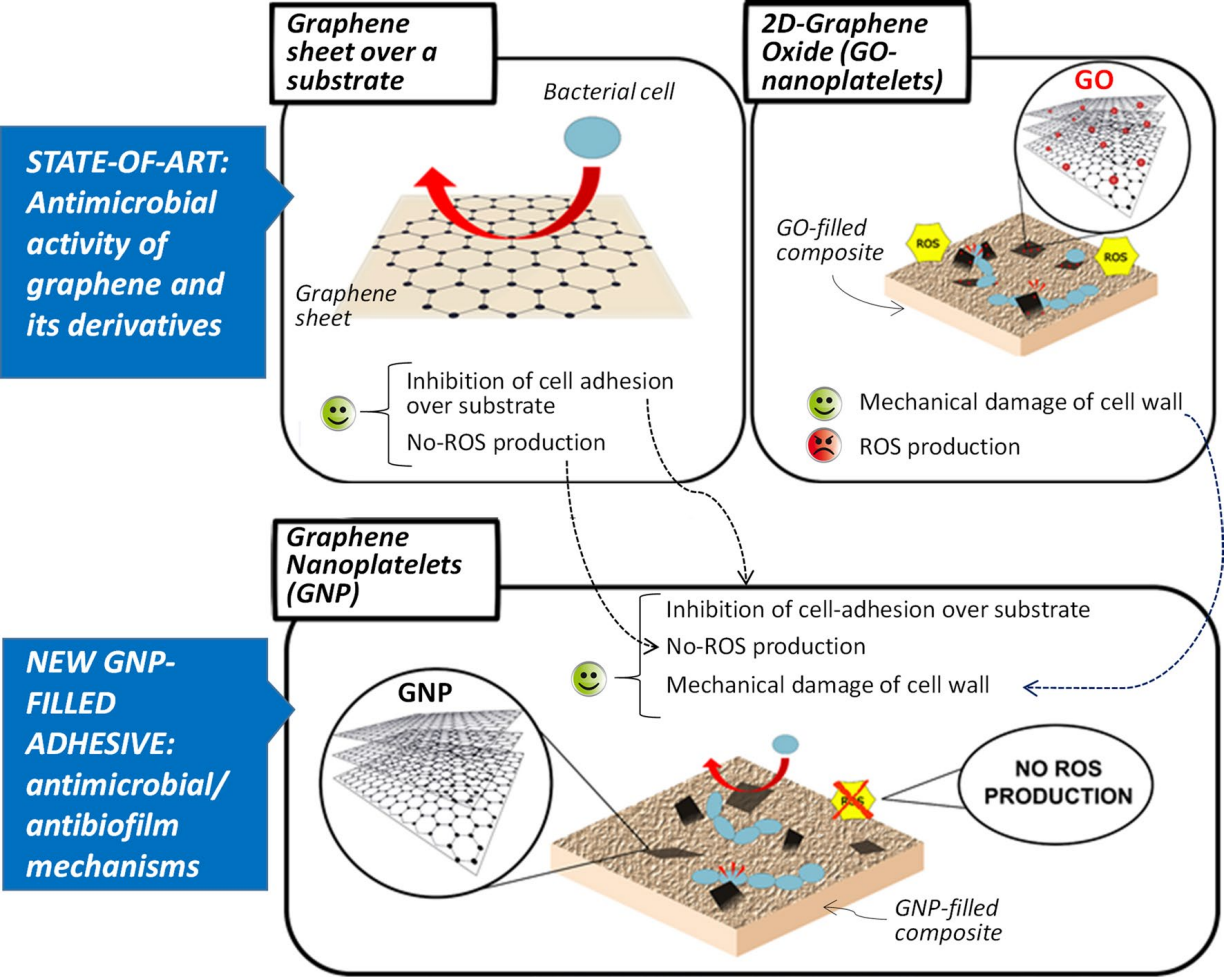
Antimicrobial and antibiofilm action mechanism of the experimental adhesive developed in this work. Graphene is known to be an anti-adhesion material. GO are well-known antimicrobial materials thanks to the shape and to the presence of oxygen containing functional groups that increase its hydrophilicity and allow ROS production. GNPs possess biocidal properties typical of 2D-shaped graphene based materials and anti-adhesion properties typical of graphene thanks to the absence of basal plane functional groups. Moreover, GNPs do not produce ROS due to the absence of oxygen reacting species, and consequently are characterized by a much lower potential of cytotoxicity when compared with GOs

The main idea behind this work is to develop a GNP-filled polymer composite that enables to combine (as sketched in Fig. 10) the anti-adhesion properties of graphene towards *S. mutans* biofilm, with the antimicrobial activity of GNPs, without producing a surplus of reactive oxygen species (ROS), which are correlated to higher cytotoxicity [35, 36, 38].

The GNPs produced by following the proposed protocol are highly reduced as confirmed by FTIR [46] and XPS [51] analysis, resulting in an anti-biofilm behaviour and no expected ROS production. Indeed, no oxidative stress was highlighted in biofilm cells grown on A02 samples in comparison with control adhesive. ROS are not produced by pristine or by highly reduced graphene materials obtained from pristine graphite or graphite intercalated compounds (GICs), so that the mechanical effect is the main one responsible for their efficacy against pathogens [52, 53]. The mechanisms underlying this mechanical damage of the bacteria membrane caused by the sharp edges of 2D carbon-based nanomaterials were demonstrated in several works through theoretical simulations using the coarse grained molecular dynamics [54, 55].

Moreover, we expected that the developed experimental antimicrobial adhesive could take advantage of a killing mechanism based on the mechanical interaction between nanostructure and cell walls (characteristics of 2D-shaped carbon nanostructures) already studied in a previous work [29] and on the biofilm anti-adhesion effect demonstrated for graphene [56].

Several attempts to fight oral pathogens have been carried out by developing dental materials containing antimicrobial compounds in order to control surface biofilm formation [57, 58]. As demonstrated by crystal violet and FE-SEM analysis, a remarkable reduction of *S. mutans* mature biofilm is observed when GNPs were added to the dental adhesive. Many studies have been addressed to rapidly kill microorganisms by exploiting new antimicrobial compounds [59]. However, a promising approach is represented by the inhibition of the initial adhesion of bacteria to surfaces, required for a mature biofilm development. Nowadays, nanomaterials are attracting attention for antibiofilm strategies since they are highly effective as antimicrobials; and bacterial cells, even after 20 passages at sub-MIC concentrations, do not show resistance; a result that is very far with respect to traditional antibiotics [60]. According to FE-SEM images, in the first 3 h of growth, *S. mutans* cells colonized the whole surface of teeth covered by the commercial dental adhesive. Conversely, only a very low number of cells are present on the surface in presence of protrusive GNPs. These results demonstrate an anti-adhesion effect when fillers are at least partially exposed over the surface. Because of the anti-adhesive properties of the GNPs, after 24 h *S. mutans* was not able to form a mature biofilm on the A02 sample in contrast to the control dental adhesive, that is totally colonized. The obtained results are consistent with the one of Parra et al., who produced an anti-biofouling material demonstrating that single and few-layer graphene coatings on SiO_2_ substrates inhibits the bacterial adhesion [56]. Nevertheless, the vitality tests demonstrate that the produced experimental adhesives possess biocidal activity, mainly ascribed to the mechanical “nano-knives” effect. Thus, this work clearly demonstrate that a dental adhesive filled with GNPs can inhibit bacteria proliferation in the oral cavity.

## Conclusion

In summary, we have demonstrated that it is possible to combine the antimicrobial mechanism of graphene-based nanoplatelets and the anti-adhesion properties of 2D graphene using GNPs. This nanomaterial, used as filler in dental adhesives, significantly inhibited the adhesion and growth of *S. mutans* in vitro, inducing a mechanical action. Moreover, using rheology, FE-SEM imaging and μ-TBS we demonstrated that the mechanical performances of the experimental dental adhesive are practically identical to the control one. Therefore, we conclude that it has been possible to optimize a novel dental anti-biofilm adhesive without altering the standard adhesion properties.

This conclusion is supported through the results of the experimental study performed in this work. Quantitative crystal violet data and qualitative FE-SEM investigations demonstrate the exploitation of GNP as nano-filler in dental adhesives.
